# The draft genome of a socially polymorphic halictid bee, *Lasioglossum albipes*

**DOI:** 10.1186/gb-2013-14-12-r142

**Published:** 2013-12-20

**Authors:** Sarah D Kocher, Cai Li, Wei Yang, Hao Tan, Soojin V Yi, Xingyu Yang, Hopi E Hoekstra, Guojie Zhang, Naomi E Pierce, Douglas W Yu

**Affiliations:** 1 Department of Organismic and Evolutionary Biology, Museum of Comparative Zoology, Harvard University, 26 Oxford St, Cambridge, MA 02138, USA; 2 China National GeneBank, BGI-Shenzhen, Shenzen 518083, China; 3 Centre for GeoGenetics, Natural History Museum of Denmark, University of Copenhagen, Øster Voldgade 5-7, Copenhagen 1350, Denmark; 4 School of Biology, Georgia Institute of Technology, Atlanta, GA 30332, USA; 5 Department of Molecular and Cellular Biology, Howard Hughes Medical Institute, Harvard University, 26 Oxford St, Cambridge, MA 02138, USA; 6 Centre for Social Evolution, Department of Biology, University of Copenhagen, Universitetsparken 15, Copenhagen DK-2100, Denmark; 7 State Key Laboratory of Genetic Resources and Evolution, Kunming Institute of Zoology, Kunming, Yunnan 650223, China; 8 School of Biological Sciences, University of East Anglia, Norwich Research Park, Norwich, Norfolk NR47TJ, UK

## Abstract

**Background:**

Taxa that harbor natural phenotypic variation are ideal for ecological genomic approaches aimed at understanding how the interplay between genetic and environmental factors can lead to the evolution of complex traits. *Lasioglossum albipes* is a polymorphic halictid bee that expresses variation in social behavior among populations, and common-garden experiments have suggested that this variation is likely to have a genetic component.

**Results:**

We present the *L. albipes* genome assembly to characterize the genetic and ecological factors associated with the evolution of social behavior. The *de novo* assembly is comparable to other published social insect genomes, with an N50 scaffold length of 602 kb. Gene families unique to *L. albipes* are associated with integrin-mediated signaling and DNA-binding domains, and several appear to be expanded in this species, including the glutathione-s-transferases and the inositol monophosphatases. *L. albipes* has an intact DNA methylation system, and *in silico* analyses suggest that methylation occurs primarily in exons. Comparisons to other insect genomes indicate that genes associated with metabolism and nucleotide binding undergo accelerated evolution in the halictid lineage. Whole-genome resequencing data from one solitary and one social *L. albipes* female identify six genes that appear to be rapidly diverging between social forms, including a putative odorant receptor and a cuticular protein.

**Conclusions:**

*L. albipes* represents a novel genetic model system for understanding the evolution of social behavior. It represents the first published genome sequence of a primitively social insect, thereby facilitating comparative genomic studies across the Hymenoptera as a whole.

## Background

Social behavior holds special distinction in evolutionary biology because it represents a major transition from an individual to a coordinated group [1]. Despite this additional layer of complexity, the same genetic and genomic methods used to study complex behaviors in model systems can be applied to the study of sociality.

One such approach is to combine genetic and ecological studies to understand how genes and the environment shape the striking diversity of behaviors that occur within and between species. Taxa harboring natural variation in a trait of interest - whether it be morphological, physiological, or behavioral - are ideal because they enable quantitative and population genetic studies to elucidate some of the underlying genetic components [2,3]. Over the past few years, this approach has helped to illuminate some of the genetic and ecological factors associated with repeated evolution of both morphological and behavioral traits: examples include benthic and limnetic forms in sticklebacks [4,5], coat color in mice [6,7], mimicry rings in *Heliconius* butterflies [8], and song performance in crickets [9,10].

Halictid bees (Hymenoptera: Halictidae) or ‘sweat bees’ are small- to medium-sized bees with a cosmopolitan distribution and over 4,000 described species. They are mass provisioners and pollen feeders, and nest primarily in the ground. Most species are solitary, but many are primitively eusocial (as defined by [11]). These species produce colonies composed of a facultatively sterile worker caste and only one or a few reproductive individuals that are not morphologically distinguishable from each other [11,12].

Halictids are particularly useful models for behavior because they harbor extensive variation in social behavior both within and between species [13,14]. Within the Halictidae, eusociality has evolved at least twice, with many subsequent reversions [15,16]. The origins of eusociality in this group are relatively young (approximately 22 to 35 millions of years ago; [15,16]), and perhaps because of this, a great deal of variation in social behavior exists, ranging from solitary to communal to eusocial (reviewed in [13,14]). Interestingly, some of these species are socially polymorphic, and females are capable of producing either solitary or social nests. These social polymorphisms can occur across geographical gradients or even within populations [17-20].

One species in particular represents an ideal system for exploring the genetic mechanisms underlying social behavior: *Lasioglossum albipes*. This socially polymorphic species is solitary in inland localities in France and Germany, but eusocial in southwestern France where the climate is warmer and nests are initiated earlier in the summer [11,18]. The life cycle of the social females is typical for a eusocial species with a univoltine life history (that is, one generation per year). Females found a nest in the spring and rear a first brood of workers that then help rear a second brood of reproductive males and females that subsequently mate and diapause through the winter to repeat the cycle the following spring. The life cycle of the solitary populations is the same except that the first, eusocial worker brood is not produced. Common-garden experiments were conducted with *L. albipes* in which both social forms were reared in the laboratory under the same conditions and also under complementary photoperiods; the typical behaviors for each population remained the same in the lab as in the field, suggesting that this behavioral polymorphism is likely to have an underlying genetic component [21]. This system thus provides an excellent model for studying the ecological and genetic factors associated with the evolution of social behavior.

Here we present the draft genome of this socially polymorphic bee*,* the first halictid for which genomic resources have been developed. We compare its genome sequence to other published insect genomes and identify a number of interesting patterns that can be tested in future studies.

## Results and discussion

### Genome sequencing and assembly

DNA was isolated and assembled from two haploid males collected from a solitary population in Leysin, Switzerland (Additional file 1). Seven paired-end libraries with insert sizes ranging from 170 bp to 10 kb were constructed and sequenced on Illumina HiSeq 2000 and GAIIx (10 kb libraries) systems. To improve scaffolds, an additional 10 kb Illumina mate-pair library was constructed from a pool of 20 females collected from multiple French and Swiss populations (Additional file 1). Before filtering, this produced 53.62 Gb of raw data. Low quality reads, reads with a high proportion of Ns or poly-A structures, overlapping paired ends, and PCR duplicates were filtered prior to assembly. Post-filtering, 39.81 Gb of raw reads remained (Table 1).

**Table 1:** Data used for genome assembly and scaffolding

**Insert size (bp)**	**Read length (bp)**	**Raw data (Gb)**	**Coverage (X)**	**Data after filtering (Gb)**	**Coverage (x)**	**GC content (%)**
200	100	8.28	19.90	7.11	17.09	40.25
500	100	14.36	34.51	9.64	23.17	39.85
800	100	8.06	19.36	5.74	13.80	42.12
2 kb	49	5.65	13.59	4.70	11.30	45.35
5 kb	49	6.77	16.30	5.61	13.49	45.90
10 kb	49	10.50	25.14	7.01	16.80	43.68
**Total**	-	53.62	128.80	39.81	95.65	42.86

DNA was sequenced on an Illumina HiSeq 2000 (100 bp read lengths) or on the Illumina GAIIx (49 bp reads). Libraries were constructed across a range of insert sizes, from 200 bp to 10 kb. The final assembly after filtering consisted of 39.81 Gb of data with 95× coverage of the genome.

The *L. albipes* genome size is estimated at 416 Mb. The final assembly has an N50 scaffold length of 602 kb and a total length of 350.8 Mb (Table 2). The genome contains a high degree of repetitive elements, which comprise 32.71% of the final assembly (Additional file 2). Completeness of the assembly was assessed using the CEGMA pipeline [22]. Of the 248 core eukaryotic genes (CEGs), 243 were completely assembled in the *L. albipes* genome. The closest relative to *L. albipes* with a genome sequence is the honey bee, *A. mellifera*, and our results are comparable to that of the *A. mellifera* v4.5 genome assembly and the other sequenced hymenopterans (Additional file 3) ([23]).

**Table 2:** Genome assembly statistics

	
Contigs (*n*)	32,498
Largest contig (bp)	14,618
Scaffolds >1 kb (*n*)	4,377
N50 scaffolds (bp)	616,426
Scaffolds >N50 (*n*)	152
Largest scaffold (bp)	3,533,895
Predicted genes	13,448
Ultra-conserved core eukaryotic genes	97.98/100
(complete/partial, %)	

Summary statistics for final *L. albipes* genome assembly. 152 scaffolds are greater than the N50 of 616 kb, with the largest assembled scaffold containing 3.5 Mb. The genome assembly appears to be nearly complete, with 98% of all core eukaryotic genes completely assembled (complete) and 100% at least partially assembled (partial). The official gene set contains 13,448 predicted genes.

### RNA sequencing

RNA sequencing was performed on four pooled adult females collected from field sites in France and Switzerland (Additional file 1). RNA was extracted and a 2 × 100 paired-end library was sequenced on an Illumina HiSeq 2000. The resulting 35,207,669 reads were mapped back to the reference genome (approximately 230× coverage assuming a 30 Mb transcriptome) using Tophat [24], and 23,308 transcript models were generated using Cufflinks [25]. These data were incorporated into the gene annotation pipeline (see below for details) to refine gene annotation.

### Gene annotation

**Figure 1:**
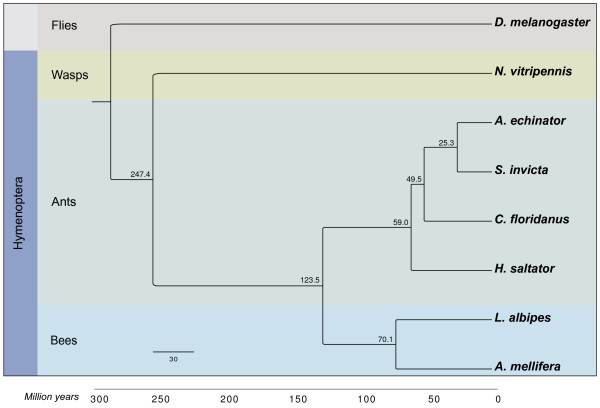
**Phylogenetic placement of *L. albipes.*** Four-fold degenerate sites were used to reconstruct the phylogeny of eight sequenced insect genomes. Numbers at the nodes represent divergence times estimated with the ‘mcmc’ package in PAML [26]. *L. albipes* and *A. mellifera* diverged approximately 70 million years ago.

A combination of RNA-sequencing, *de novo,* and homology-based gene predictions generated an official gene set including 13,448 predicted genes (Additional files 4 and 5). Orthology was assigned using reciprocal best BLASTs (Additional file 6). Non-coding RNAs (ncRNAs) are summarized in Additional file 7. Treefam was used to cluster genes into 9,614 gene families using information from six additional hymenopterans (the honey bee, *Apis mellifera,* four ants, *Acromyrmex echinatior, Solenopsis invicta, Camponotus floridanus, Harpegnathos saltator,* and the parasitoid wasp, *Nasonia vitripennis),* plus one additional insect as an outgroup to the Hymenoptera (the dipteran fruit fly*, Drosophila melanogaster*). Multiple alignments of protein sequences were generated for each gene family across these eight insect species, and the four-fold degenerate sites were used to reconstruct the phylogeny (Figure 1).

**Figure 2:**
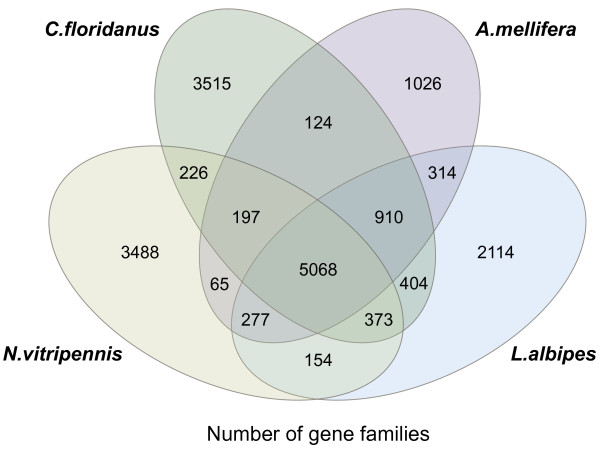
**Overlap among gene families for four 4 hymenopteran species.** Numbers indicate the gene families in each comparison. A total of 5,068 gene families are shared among all four species.

There were 5,068 gene families shared among all four hymenopteran species, and 1,981 predicted genes that appear to be unique to the *L. albipes* lineage (Figure 2). Functional enrichment analyses were conducted using chi-square and Fisher Exact tests (for small sample sizes) to calculate significance, and an FDR correction was applied to account for multiple testing [27]. The gene ontology (GO) and InterPro protein domain (IPR) enrichment results for *L. albipes*-specific genes are listed in Additional files 8 and 9. Among these 1,981 unique genes, many are associated with the integrin-mediated signaling pathway (*P* <0.001) and have an over-representation of protein domains associated with nucleases (*P* <0.02), MADF/BESS domains (*P* <0.0001), and ankyrin and PRANC domains (*P* <0.0001).

### Gene family expansion

**Figure 3:**
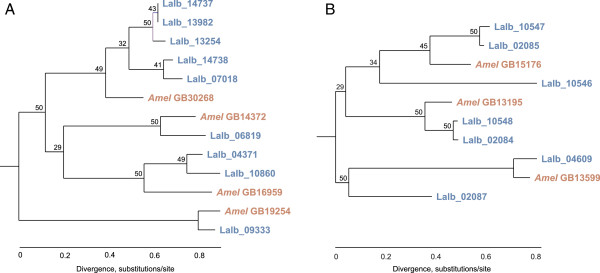
**Gene family expansions in *L. albipes. ***Two gene families appear to have expanded in the *L. albipes* lineage. Trees were calculated using maximum-likelihood. **(A)** Glutathione-S-transferase gene family. Nine homologs of this family were identified in *L. albipes* (blue branches) in contrast to only four known homologs in *A. mellifera* (orange branches). **(B)** Inositol monophosphatase gene family. Seven members of this family are found in the *L. albipes* genome (blue branches), in contrast to three homologs in *A. mellifera* (orange branches)*.*

Two notable gene families appear to be expanded in the *L. albipes* lineage: glutathione-S-transferases (GSTs) and the inositol monophosphatases (IMPs) (Figure 3). The GST gene family is associated with the metabolism of secondary compounds and insecticides in insects. Specifically, these enzymes catalyze a reaction between glutathione and these compounds, making them more soluble and easier to excrete. This gene family also plays an important role in intracellular transport, hormone biosynthesis, and protection against oxidative stress [28]. The *L. albipes* genome contains nine members of this gene family, in contrast to four genes in *A. mellifera*. Only two of the four *A. mellifera* orthologs appear to be duplicated in *L. albipes* (Figure 3A). The inositol monophosphatase gene family is a group of dephosphorylating enzymes used to free *myo-*inositol in eukaryotic taxa [29] and is associated with lipid metabolism. *L. albipes* has seven genes in this family, while *A. mellifera* has only three (Figure 3B). The expansions of the IMP gene family in *L. albipes* may reflect the life history of this species where, unlike *A. mellifera*, foundresses must undergo diapause as adults prior to founding a new nest in the spring and as a result, efficient nutrient storage and lipid metabolism may be particularly crucial to survival and reproduction.

We also characterized over- and under-represented IPR domains in the *L. albipes* gene set in comparison to *A. mellifera*. IPR domains with >2-fold difference between *L. albipes* and *A. mellifera* were considered as over- or under-represented. There were 92 IPR domains overrepresented in *L. albipes* (Additional file 10), including some associated with the expanded gene families discussed above, such as IPR017933 and IPR000760. Additionally, the MADF domain (IPR006578) has 42 copies in *L. albipes* but only nine in *A. mellifera*. This domain is associated with transcription factor *Adf-1* in *Drosophila*, and is known to play a role in the regulation of alcohol dehydrogenase expression [30]. There are also several fatty acid-related domains over-represented in *L. albipes* (IPR015876, IPR005804 and IPR020842). Previous studies in *H. saltator* found expression of a fatty acid synthase to be upregulated in reproductive females relative to workers [31].

### Gene family contraction

Several genes appear to be lost in *L. albipes* but present in the six other sequenced hymenopterans (Additional file 11), and 36 IPR domains are under-represented in the *L. albipes* gene set when compared with *A. mellifera* (Additional file 12)*.* One under-represented domain of interest is IPR017996 (‘Major Royal Jelly’ protein (MRJP)). Yellow and royal-jelly-like proteins control expression of genes affecting cuticular pigmentation, development, sexual maturation and behavior [32], and are associated with caste determination in the honey bee [33]. We manually curated the *yellow* and MRJP gene families, and found 10 *yellow* genes in *L. albipes*, the same number as in *A. mellifera*. In contrast, only two credible MRJP genes were found in *L. albipes*, similar to the number found in the ant species *C. floridanus* and *H. saltator*. The ML tree of yellow genes and MRJP genes is shown in Additional file 13.

### DNA methyltransferases

Epigenetic mechanisms can play an important role in gene regulation and phenotypic plasticity. DNA methylation appears to be one of the key mechanisms underlying transgenerational epigenetic effects. DNA methylation is widespread across Hymenoptera [34] and has been implicated in caste differentiation in honey bees and ants [31,35,36].

DNA methyltransferases (DNMTs) are the genes that perform DNA methylation; all share a conserved catalytic domain, suggesting a common and ancient origin [37]. Studies of mammalian systems have established that different DNMTs undertake distinct functions (reviewed in [38]). For example, human genomes contain two DNMT1s, one DNMT2, and one DNMT3 (DNMT3a/b). DNMT1 is responsible for maintaining the patterns of DNA methylation between DNA replications and is referred to as the ‘*maintenance* methyltransferase’. The role of DNMT2 is still not completely resolved, but recent studies suggest that it may act as a tRNA methyltransferase. Finally, DNMT3s mediate *de novo* methylation of previously unmethylated cytosines.

We investigated whether *L. albipes* exons contain a complete repertoire of putative DNMTs. Using a homology-based search (Additional files 14 and 15), we found strong evidence that the *L. albipes* genome encodes two putative xDNMT1s (Lalb_01810 and Lalb_06290), one putative DNMT2 (Lalb_08279), and one putative DNMT3 (Lalb_11571). These results demonstrate that *L. albipes* appears to have an intact DNA methylation system.

### CpG content in and around genes

DNA methylation patterns show a high degree of conservation across insect taxa. Methylation appears to occur primarily in exons and in 5′ UTRs (untranslated regions) and has been implicated in alternative splicing [39-42]. In animal genomes, DNA methylation occurs primarily at CpG dinucleotides (cytosine followed by guanine). Because methylated cytosines undergo frequent deamination and tend to mutate to thymines, methylated CpG regions tend to have lower frequencies of CpG dinucleotides [43]. To investigate the presence of DNA methylation and its influence, we examined normalized CpG content (CpG O/E) across different genomic regions in *L. albipes*. Regions with lower CpG content than expected are interpreted as a signal of methylation [44].

**Figure 4:**
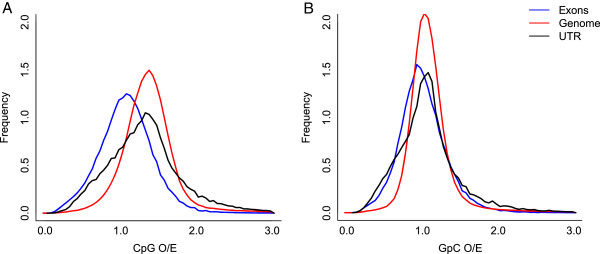
**CpG Content in *L. albipes. *(A)** Exons and mRNAs have lower CpG than expected, suggesting that gene bodies may be preferentially methylated in *L. albipes,* consistent with previous findings in *A. mellifera*. **(B)** GpC ratios were also calculated as a control. GpC dinucleotides have the same sequence composition as CpGs, but are not subject to biased mutation rates due to DNA methylation.

Genomic fragments in *L. albipes* have a CpG O/E of 1.58, indicating that there is an overabundance of CpG dinucleotides in this species. These results are similar to the honey bee, which has a CpG O/E of 1.67 [45]. Despite this genome-wide overabundance of CpG dinucleotides, *L. albipes* coding sequences (CDS) and gene bodies exhibit lower CpG O/E values than the genomic background (*P* <10^-160^ for both), suggesting that DNA methylation may impact CpG content in CDS (Figure 4). Furthermore, CDSs exhibit significantly lower CpG content than do gene bodies, suggesting that that DNA methylation occurs primarily in exons (Figure 4A).

Somewhat surprisingly, GpC O/E values also varied significantly between CDS and the genomic background (*P* <10^-160^), though these differences were less pronounced than they are for CpG O/E (Figure 4B). This was caused by G+C content skew in CDS, which are particularly GC-enriched in *L. albipes* (Additional file 16). Interestingly, there is a strong negative correlation between GpC O/E and GC content as well as between CpG O/E and GC content (Additional file 17). After controlling for underlying GC content variation, only CpG O/E ratios exhibit clear and substantial differences from the genomic background (Additional file 18), providing strong support for DNA methylation in *L. albipes* exons.

Based on these results, we propose that DNA methylation occurs in the *L. albipes* genome, although experimental validation will be necessary. A subset of 1,801 genes harbor extreme differences in CpG and GpC O/E values and are likely to reflect methylation in these regions (Additional file 19). The negative correlation between normalized CpG and GpC content versus GC content is unique in the *L. albipes* genome and appears to be opposite to the pattern observed in the honey bee [46], suggesting that additional, unknown evolutionary forces are acting on the nucleotide composition of *L. albipes*.

### Molecular evolution

*L. albipes* is the first bee whose genome has been characterized that does not belong to the corbiculate bees (Hymenoptera: Apidae) and, as such, represents a novel lineage for comparative studies aimed at identifying the molecular toolkit associated with the evolution of social behavior. To identify genes showing signatures of accelerated evolution in *L. albipes* (halictid bees) and/or Apoidea (all bees), we conducted branch-specific tests in PAML [26]. We chose six species representative of the sequenced Hymenopteran genomes to perform these analyses. These taxa include two bees (*L. albipes* and *A. mellifera*)*,* two ants (*H. saltator* and *S. invicta*)*,* the parasitoid wasp, *N. vitripennis*, as an outgroup to the social Hymenoptera, and the fruit fly, *D. melanogaster,* as an outgroup to the Hymenoptera as a whole. We used the ‘branch’ model in PAML to search for signatures of accelerated evolution in focal lineages using a likelihood-ratio test (LRT) to calculate significance. Following correction for multiple testing, 615 genes showed signatures of accelerated evolution in the *L. albipes* lineage when compared to the remaining branches (FDR <0.05; Additional file 20), and 899 in Apoidea when contrasted to the remaining branches (FDR <0.05; Additional file 21).

**Figure 5:**
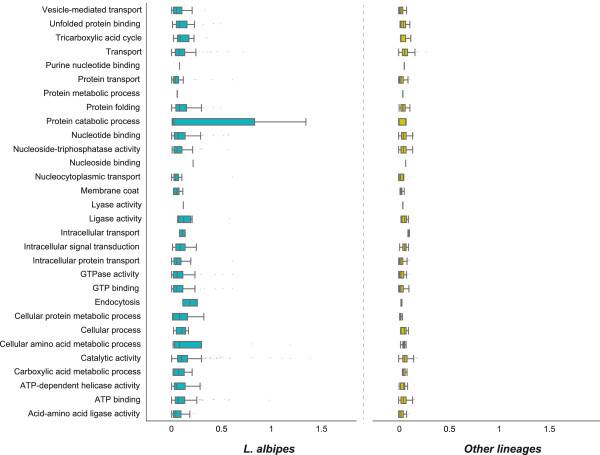
**Accelerated evolution in *L. albipes. ***Gene ontology categories with a significant over-representation of genes showing signatures of accelerated evolution on the *L. albipes* branch (FDR <0.05) are specified on the y-axis. ω (dN/dS) values are on the x-axis with the scale ranging from ω =0 to ω =2. Blue boxplots indicate the range of ω for the genes associated with each GO term on the *L. albipes* branch. The yellow boxplots indicate the range of ω values for these genes in the remaining branches.

Functional enrichment analyses for these genes are summarized in Additional files 22, 23, and 24. In general, genes associated with carboxylic acid metabolism, cell signaling, and protein transport appear to be subject to accelerated evolution in *L. albipes* relative to the other species examined (Figure 5). Heat shock proteins (Additional file 23) are also evolving more quickly in the halictid lineage. This gene family is known to play a key role in diapause in a number of insect species [47] and is potentially interesting given the population-level correlation between sociality and microclimate in *L. albipes*[11,18]. Furthermore, many halictid species exhibit strong behavioral plasticity in response to local environment [17-20]. It is possible that the accelerated evolution of heat shock proteins may reflect this group’s ability to determine the behaviorally appropriate response to environmental conditions.

### Differences between social forms

To look for gross genetic differences between social forms, individual females from one solitary and one social population were sequenced to approximately 15× coverage. Remapping of these reads to the reference genome revealed that these individuals vary as much from each other as they do from the reference sequence, with 499,486 SNPs unique to the solitary female (compared to the social female and the reference sequence) and 493,579 SNPs unique to the social female (compared to the reference and solitary female). Per-site Watterson’s Θ_W_ was calculated using four-fold degenerate nucleotides, and is estimated at 0.003. Comparisons with *A. mellifera* (Θ_W_ approximately 0.09 for non-coding sites) ([48]) suggest potentially reduced level of genetic diversity of *L. albipes* compared to the honey bee.

The numbers of synonymous (Ks) and non-synonymous (Ka) substitutions and their ratio (Ka/Ks) were calculated for each gene to search for loci showing signatures of accelerated evolution between social forms. Sliding window analyses did not reveal any large genomic regions that appear to be differentiated between social forms, but six genes had Ka/Ks values >1, indicating that these sequences could be diverging rapidly between these two populations (FDR <0.1; Additional file 25). Two of these genes encode a putative odorant receptor (Lalb_14702) and a cuticular protein similar to *apidermin-3* (Lalb_0725) ([49]), perhaps indicating that differences in chemical signaling and/or pheromone production are associated with shifts in sociality. The remaining genes include: a metalloendopeptidase similar to *neprilysin 1*, which is associated with modulation of neurotransmitter levels and expressed in the brain mushroom bodies in *Drosophila*[50], and a receptor-type tyrosine-protein phosphatase associated with the regulation of axon guidance and also with autism in humans [51]*.* These genes provide an interesting set of candidates for further examination, but given that these genetic differences were characterized between two individuals, further work examining multiple individuals from a number of solitary and social populations is needed to fully characterize signatures of selection between the solitary and social behavioral forms within *L. albipes*.

## Conclusions

Its intraspecific behavioral variation makes *L. albipes* ideal for population and ecological genomic studies to characterize the underlying genetic components associated with solitary and social behavior. Our results suggest that mechanisms associated with DNA methylation and nutrient storage may play a role in modulating social behavior in this species, and future research will examine these pathways in more detail. The addition of *L. albipes* to the published hymenopteran genomes establishes a framework for further phylogenetic comparisons that we can use to investigate forces that have shaped the evolution of social behavior.

## Materials and methods

### Sample preparation and library construction

#### Genomic DNA

Whole bodies were first rinsed in ethanol then ground in liquid nitrogen to facilitate extraction of genetic material. DNA extractions were performed using a Qiagen Genomic-tip 20/G kit (Valencia, CA, USA) and standard protocol. Genomic DNA extracted from the samples Albi-2 and Albi-3 was used to generate non-amplified DNA libraries of 200 and 500 bp. To obtain sufficient genomic DNA, we performed a multiple displacement amplification on the Albi-2 sample using the REPLI-g Midi kit (Qiagen, Valencia, CA, USA) prior to library construction. This may have contributed to lower GC content within these libraries, and as such, additional 200, 500, and 800 bp libraries were constructed from unamplified genomic DNA from the Albi-3 sample. To improve genome assembly, genomic DNA from 20 pooled females was also used to construct an amplification-free 10 kb library.

For library construction, DNA was sheared to fragments of size 200 to 500 bp. Ends were repaired, A-tailed, and ligated to paired-end adapters (Illumina, San Diego, CA, USA). A size selection was then performed by agarose gel, and fragments were amplified with LM-PCR. Long-insert libraries were constructed by shearing genomic DNA to the appropriate insert size with nebulization (2 kb library) or HydroShear (5 kb and 10 kb libraries; Covaris, Woburn, MA, USA). Fragments were end-repaired with biotinylated nucleotide analogues (Illumina, San Diego, CA, USA), and size-selected fragments (2 kb, 5 kb, and 10 kb) were circularized via intramolecular ligation, sheared to 500 bp with Adaptive Focused Acoustic (Covaris, Woburn, MA, USA), and purified on magnetic beads (Invitrogen, Carlsbad, CA, USA). These purified fragments were then end-repaired, A-tailed, and ligated to paired end adapters (Illumina). A final size selection step and LM-PCR purification was conducted prior to sequencing.

#### Total RNA

For transcriptome sequencing, RNA was extracted from four individual females using a Qiagen RNeasy extraction kit (Valencia, CA, USA) and standard protocol. RNA was then pooled and cDNA libraries constructed.

First strand cDNA was synthesized using random hexamers and Superscript II reverse transcriptase (Invitrogen, Carlsbad, CA, USA). *E. coli* DNA PolI (Invitrogen) was used for second strand synthesis, and double stranded cDNA was then purified using the Qiaquick PCR purification kit (Qiagen, Valencia, CA, USA). Purified cDNA was sheared to 100 to 500 bp fragments with a nebulizer (Invitrogen), end-repaired, and a 3′ dA overhang added to the ends. Illumina adapters were ligated to the cDNA and size selected to 200 ± 20 bp on an agarose gel. Fifteen cycles of PCR amplification were conducted prior to sequencing. Gel-purification of 18 to 30 nt RNA was used for smRNA-seq. 5′ and 3′ Illumina RNA adapters were ligated to these fragments, and products were size-selected on a denaturing polyacrylamide gel. These purified products were then reverse transcribed with small RNA RT primers and amplified with small RNA PCR primers 1 and 2 (Illumina) with 15 cycles of PCR prior to sequencing.

### Genome assembly

SOAPdenovo [52] was used for genome assembly. We constructed a de Bruijn graph using the parameter ‘-K 47’. Then, default parameters were used to simplify the graph and generate contigs by removing tips, merging bubbles and solving repeats. All sequenced reads were then realigned onto the contig sequences with the parameters: ‘-k 47 -f’. Finally, scaffolds were constructed by weighting the rates of consistent and conflicting paired-end relationships with parameter: ‘-F -u’. All usable reads were realigned to contigs and paired-end information was used to assemble scaffolds and close gaps. Raw sequencing reads were mapped back to the scaffolds using SOAPaligner [53] with options ‘-m 0 -x 1000 -v 5’ for 200 bp, 500 bp, and 800 bp libraries, ‘-m 0 -x 10000 -R -v 3’ for 2 kb and 5 kb libraries, and ‘-m 0 -x 20000 -R -v 3’ for the 10 kb library. The results were used to check for GC bias in the sequencing data. Potential contaminants were filtered using BLASTN to align all assembled sequences against the NCBI nt database (version: 20110312), and sequences with best hits to bacterial or fungal sequences were removed from the assembly and excluded in downstream analyses. Completeness of the assembly was assessed using the CEGMA pipeline [22].

### Repeat annotation

Known transposable elements (TEs) were identified with RepeatMasker (version 3.2.6) ([54]) using the Repbase TE library (v. 15.02) ([55]) and default parameters. A consensus sequence for each repeat family was generated and used as the library in RepeatMasker to identify additional high and medium copy repeats (>10 copies) in the genome assembly. Tandem repeats were predicted using TRF [56], with parameters set to ‘Match = 2, Mismatch = 7, Delta = 7, PM = 80, PI = 10, Minscore = 50, and MaxPeriod = 12’. In total, 107.29 Mb of repetitive sequence were identified, comprising 32.71% of the assembled genome (Additional file 2).

### Protein-coding gene annotation

Three different methods were used to predict protein-coding genes: (a) homology-based methods, (b) *de novo* prediction*,* and (c) RNA sequencing. Gene sets from four species (*C. elegans, D. melanogaster, H. saltator,* and *A. mellifera*) were used for homology-based predictions, one species at a time. TBLASTN was used to search the non-redundant protein sequences of each gene set with an E-value <1e-5. The best hit was then selected, and regions with homologous blocks shorter than 50% of the query protein were excluded. We then used GENEWISE (v. 20.0) ([57]) to generate the gene structures. Homology-based repeats were masked in the genome, and AUGUSTUS [58] and SNAP [59] were used for *de novo* gene prediction. Parameters were trained using 2,682 high quality genes with intact ORFs based on homology to *A. mellifera*. Evidence derived from homology-based predictions (4 sets) and *de novo* predictions (2 sets) were then integrated in GLEAN to generate a consensus gene set. Based on these analyses, 22,068 genes passed the GLEAN criteria.

To improve gene annotation, RNA sequencing was performed on four pooled adult females collected from field sites in France and Switzerland (Additional file 1). Reads were mapped to the current genome assembly using Tophat [24], and then Cufflinks [25] was used to assemble the mapped reads into transcripts with the following parameters for Tophat: ‘-r 20 -mate-std-dev 10 -I 10000’ , and for Cufflinks: ‘-I 50000’. ORFs were predicted in assembled transcripts using BGI’s in-house pipeline, CCG. CCG also integrated the gene models from GLEAN with the transcript-based models in Cufflinks to generate an improved gene set.

Finally, manual curation and visual screening was performed to refine the final gene set. The transcript-based gene models with intact ORFs that had no overlap with the CCG gene set were added. If a transcript-based gene model with an intact ORF covered more than one homology-based gene, the homology-based gene would be replaced by the transcript-based gene model. Gene models supported by more than homology prediction but that had no overlapping genes in the gene set were added. Gene models predicted to be transposable element-related (based on IPRscan and Swiss-Prot annotation) were removed. Furthermore, genes of particular interest (for example, the expanded and contracted gene families) were manually checked. A final gene set of 13,448 genes was used for downstream analysis.

### Functional annotation

Protein function was assigned using BLASTP best hits to the Swiss-Prot database (E-value <1e-5). Gene motifs and domains were determined using by InterProScan [60] against the InterPro database [61]. Gene Ontology (GO) annotations for each gene were obtained from the corresponding InterPro entry. The KEGG orthology [62] annotation was done by KAAS online server [63] using the SBH method. The pathways in which each gene might be involved were derived from the best KO hit. The statistics of functional annotation is provided in Additional file 11. All functional enrichment analyses were conducted using custom scripts.

### ncRNA annotation

ncRNAs were predicted using INFERNAL [64] and tRNAscan-SE [65]. Four types of ncRNA were annotated: microRNA (miRNA), transfer RNA (tRNA), ribosomal RNA (rRNA), and small nuclear RNA(snRNA). tRNA genes were predicted by tRNAscan-SE with eukaryote parameters. rRNA fragments were identified by aligning the rRNA template sequences from invertebrate animal using BLASTN with an E-value <1E-5. miRNA and snRNA genes were predicted by INFERNAL using the Rfam database (release 9.1). To accelerate the speed, a rough filtering was performed before INFERNAL, by Blastn against the Rfam sequence database with an E-value cutoff of 1. Additional file 12 summarizes the statistics of ncRNA annotation.

### Construction of gene families

To gain insight into the evolution of *L. albipes* gene families, we used Treefam [66] to cluster protein-coding genes from eight insect species (*A. echinatior, S. invicta, C. floridanus, H. saltator, L. albipes, A. mellifera, N. vitripennis, and D. melanogaster*) into gene families. Only the longest transcript isoform was used for each gene. BLASTP (with E-value <1e-5) was performed against a blast database including protein-coding sequences for all species. Graph based methods were used to join fragmental alignments for each gene using the solar package in Treefam. We assigned a connection (edge) between two nodes (genes) if more than one-third of the region was aligned in both genes. A H-score ranging from 0 to 100 was used to weigh the similarity (edge). For two genes G1 and G2, the H-score was defined as score (G1G2)/max (score(G1G1), score (G2G2)), (score = BLAST raw score). We used the average distance for the hierarchical clustering algorithm, requiring the minimum edge weight (H-score) to be larger than 10, the minimum edge density (total number of edges/theoretical number of edges) to be larger than 0.34. After clustering, we generated multiple alignments of protein sequences for each gene family using MUSCLE [67], and converted the protein alignments to CDS alignments. A Venn diagram including the Hymenopteran species is shown as Additional file 13.

### Phylogeny construction

Four-fold degenerate sites were used from the single-copy gene family alignments, and used to reconstruct the phylogeny of these eight species in MrBayes [68] with default parameters. Divergence times of the nodes were inferred using the ‘mcmctree’ package in PAML [26].

### Gene family expansion and contraction

We used CAFÉ [69] to identify gene family expansions and contractions in *L. albipes*. This revealed two gene family expansions: glutathione-S-transferases (Additional file 15), and inositol monophosphatase (Additional file 16). Maximum-likelihood (ML) trees of the expanded families were constructed with PhyML [70]. Roots of the trees were determined using the ‘root’ function in TreeBest [71].

### Genes specific to *L. albipes*

We performed functional enrichment analyses with custom scripts using chi-squares and Fisher Exact tests (for small sample sizes) to calculate statistical significance. We then performed an FDR [27] correction to account for multiple testing. The GO/IPR/KEGG enrichment results for *L. albipes* specific genes are listed in Additional file 7: Tables S7, Additional file 8: Table S8 and Additional file 9: Table S9.

### Gene loss

A gene was considered to be lost if it was absent in *L. albipes* but present in the six other hymenopteran insects (*A. echinatior, S. invicta, C. floridanus, H. saltator, A. mellifera* and *N. vitripennis*)*.* To ensure these genes were not due to incorrect clustering or uncompleted annotation, we realigned these genes against the genome assembly. Genes that failed to pass the previous gene prediction criteria, but that had strong evidence of homology (Genewise score > = 70) and were supported by expression data (average coverage depth by RNA-seq data > 1) were reintegrated into the final gene set. Following this, 30 families were found to be lost in *L. albipes.* Each of the 30 families has only one homolog in *A. mellifera* (Additional files 11 and 12).

### Ortholog identification

We used the other 11 species, including nine Hymenoptera species *A. mellifera*, *N. vitripennis*, *H. saltator*, *C. floridanus*, *A. echinatior*, *S. invicta P. barbatus A. cephalotes* and *L. humile*, as well as *D. melanogaster* and *H. sapiens* to identify ortholog groups with *L. albipes* with BLASTP (Additional file 24). For each gene set, we performed all-against-all blasts. Then, we filtered the results by requiring the aligned rates of both target and required that the query must be >50%. We used the reciprocal best hit (RBH) of Blast score to determine orthologs.

### Characterization of DNA methyltransferases

We performed a BLASTP search against the human (*Homo sapiens*), honeybee (*A. mellifera*), chicken (*Gallus gallus*), and *Nasonia* (*N. vitripennis*) *dnmt*s using all *L. albipes* proteins as queries. Then potential *L. albipes* homologs and their query sequences were used to construct a phylogenetic tree using maximum-likelihood using the JTT model.

### CpG content

We calculated the normalized CpG content in four types of sequences: exons, introns, UTR, and whole-genome genomic fragments of 1,000 bp. These values were estimated using the formula:

$$\mathrm{Cp}G_{O/E}=\frac{P_{\text{CpG}}}{P_{C}*P_{G}}$$

where ***P***_*CpG*_, ***P***_*c*_, and ***P***_*G*_ represent the frequencies of CpG dinucleotides, C nucleotides, and G nucleotides, respectively, estimated from each genomic fragment. Because GpC dinucleotides have the same sequence composition as CpG dinucleotides, but are not subject to DNA methylation, this calculation represents a negative control. See [44] for further methodological details.

To account for the strong negative correlation between G+C content and both CpG and GpC O/E values, we divided genes and genomic fragments into five groups according to their G+C content, specifically, G+C < 0.35, 0.35 ≤ G+C < 0.45, 0.45 ≤ G+C < 0.5, 0.5 ≤ G+C < 0.55 and G+C ≥ 0.55. This allowed us to compare CpG O/E and GpC O/E of different genomic regions while accounting for G+C content.

Potentially methylated CDS are defined as those with significantly lower CpG O/E than the genomic background while exhibiting not significantly different GpC O/E than the genomic background. We performed a permutation test to determine whether CpG O/E of a specific gene is significantly lower than genome background. *P* values were determined as the ratio of 1,000 bp genomes fragments whose CpG O/E (GpC O/E) is lower than the focal fragment. The *P* values were then FDR-adjusted for multiple testing. With FDR <0.2, we observed 1,814 CDs with significantly lower CpG O/E while only 27 CDs with significant lower GpC O/E. Among the 1,814 CDs, 13 of them exhibit both significantly lower CpG O/E and GpC O/E. After discarding those 13 CDs, we considered the remaining 1,801 CDs as potentially methylated.

### Molecular evolution

We used PAML to search for genes showing signatures of accelerated evolution in (a) the *L. albipes* lineage or (b) the Apoidea lineage. We chose six species (*L. albipes, A. mellifera, H. saltator, S.invicta, N. vitripennis,* and *D. melanogaster*) to perform the accelerated evolution analyses. First, the phylogenetic tree was inferred from the four-fold degenerate sites of orthologous groups in the six species. To do LRTs with PAML, we ran one-rate branch models (‘model = 0’ in PAML control file) and two-rate branch models (‘model = 2’ in PAML control file). Two kinds of two-rate branch models were run: one for the *L. albipes* lineage, the other for the Apoidea lineage. Other parameters set in the PAML control file were ‘codonfreq = 2, kappa = 2.5, initial omega = 0.2, and fix alpha = 1’. *P* values were FDR-adjusted with a cutoff of 0.05. Functional enrichment analyses were then conducted for the genes that were found under accelerated evolution (Additional files 14, 15, and 16).

### Individual resequencing data

Individual females from a solitary and social population were sequenced to approximately 15× coverage on an Illumina HiSeq (2 × 150, paired end) in order to look for large genetic differences between social forms. DNA from each female was extracted using the AutoGen DNA extraction kit (AutoGen, Holliston, MA, USA). Whole bodies were first rinsed in ethanol then ground in liquid nitrogen to facilitate extraction of genetic material. DNA was sheared to approximately 400 bp using HydroShear (Covaris, Woburn, MA, USA). Libraries were constructed using the PrepX ILM DNA Kit for the Apollo 324 system (IntegenX, Pleasanton, CA, USA), and sequenced with a Rapid Run (2 × 150 bp) on an Illumina HiSeq 2500.

Reads were quality checked using FastQC [72] and were then mapped back to the reference genome using Stampy [73] using default parameters. Variants were called following the best practices in the Genome Analysis Toolkit v2.7.2 (Broad Institute, Cambridge, MA, USA) and included a local realignment step and variant calling with Haplotype Caller. SNPs were filtered using the following parameters: QD <2.0, FS >60.0, MQ <40.0, HaplotypeScore >13.0, MappingQualityRankSum <-12.5, and ReadPosRankSum <-8.0. Nucleotide diversity was calculated using vcftools [74], and Θ_W_ was estimated from four-fold degenerate nucleotides using custom scripts (available upon request). Ka/Ks calculations were performed using KaKs calculator using the YN model averaging method [75], and FDR-corrections were performed with the *P* adjust package in R (v2.12). Sliding windows were calculated in 100 kb increments across the genome to look for tracts with a high degree of differentiation. Alignments of significant genes were manually checked; one gene (Lalb_07521) was excluded from the list due to uncertainty in read mapping (possibly from a paralogous gene).

### Data access

This whole genome shotgun project has been deposited at DDBJ/EMBL/GenBankunder the accession SRP016091. The version described in this paper is the first version, SRP016091. The RNA sequencing reads have been deposited in the short read archive under the accession SRX190462, and the individual resequencing data have been deposited in the short read archive under the accessions SAMN02429130 and SAMN02429131.

## Abbreviations

CDS: Coding sequences; CEG: Core eukaryotic genes; DNMT: DNA methyltransferase; GO: Gene ontology; GST: Glutathione-s-transferase; IMP: Inositol monophosphatase; IPR: InterPro; MRJP: Major royal jelly proteins.

## Competing interests

The authors declare no competing interests.

## Authors’ contributions

SDK, DWY, NEP, and HEH designed the study. SDK procured and prepared the samples, generated and analyzed the individual resequencing data, and wrote the paper. LC and GZ generated the sequencing data. LC, WY, and HT performed the genome assembly and annotation and molecular evolution analyses. XY and SVY performed the CpG O/E and DNMT analyses. DWY, NEP, and HEH contributed resources and contributed in drafting the manuscript. All authors read and approved the final manuscript.

## Authors’ information

Naomi E. Pierce and Douglas W. Yu are co-seniors authors.

## Supplementary Material

Additional file 1 **Sample information.** Sample collection data for specimens used in genome and transcriptome sequencing. Sample names, sex, collection dates, region, and GPS coordinates are specified, as well as the libraries each specimen was used to construct. Click here for file

Additional file 2 **Repeats in the genome.** Repeat annotation was conducting using RepeatMasker. The overlaps between repeats have been excluded before the calculation of the total size. The length and percent of the genome comprised by each repeat is included. Click here for file

Additional file 3 **Genome assembly comparisons.** Comparison of genome assemblies for sequenced hymenopteran species. *L. albipes* is highly comparable to these other sequenced species. Click here for file

Additional file 4 **Gene prediction statistics.** Gene prediction relied on three strategies: *de novo* prediction, homology-based approaches using four well-annotated genomes, and RNA sequencing (CCG). Statistics indicate the number of genes annotated with each method, the average transcript and coding sequence (CDS) lengths, the average number of exons per gene, and the average exon and intron lengths. Click here for file

Additional file 5 **Gene predictions in comparison to other sequenced insect genomes.** Comparisons of coding sequence (CDS), mRNA, exon, and intron length were conducted across five arthropod genomes. Amel: *Apis mellifera,* Cele: *Caenorhabditis elegans,* Dmel: *Drosophila melanogaster,* Hsal: *Harpegnathos saltator*, Lalb: *Lasioglossum albipes.* Click here for file

Additional file 6 **Orthology between *L. albipes *and other species.** The top row includes the number of genes annotated in the current *L. albipes* assembly, and subsequent rows represent the number of orthologs in *L. albipes* in comparison with each named species, all sequenced ants (*H. saltator, C. floridanus, A. echinatior, S. invicta, L. humile, P. barbatus,* and *A. cephalotes*), and all sequenced Hymenoptera (all ants plus *A. mellifera* and *N. vitripennis*). Click here for file

Additional file 7 **Non-coding RNA genes in the genome.** Annotated ncRNA summary statistics. The average length of miRNA is for the predicted *precursor miRNA. The number of copies annotated in the genome, their average length in basepairs, summed total length, and the percentage of the genome comprised by each element are included.* Click here for file

Additional file 8 **GO enrichment in *L. albipes *specific genes.** The *P* values were adjusted by FDR and the cutoff of adjusted *P* value is 0.05. Click here for file

Additional file 9 **IPR enrichment in *L. albipes *specific genes.** The *P* values were adjusted by FDR and the cutoff of adjusted *P* value is 0.05. Click here for file

Additional file 10 **IPR domains over-represented in the *L. albipes *lineage.** The domains that have at least 10 copies are included in this table. Additional columns report the number of domains characterized in each species. Aech: *A. echinatior,* Amel: *A. mellifera*, Cflo: *C. floridanus*, Dmel: *D. melanogaster*, Hsal: *H. saltator*, Lalb: *L. albipes*, Nvit: *N. vitripennis*, Sinv: *S. invicta*. Click here for file

Additional file 11 **Putatively lost genes in *L. albipes *lineage.** Genes that appear to be lost in the *L. albipes * lineage are included in this table. The functions are derived from Swiss-Prot annotation database. *Amel* gene IDs represent the gene annotation symbol in the *Apis mellifera* genome assembly. Click here for file

Additional file 12 **IPR domains under-represented in *L. albipes *lineage.** IPR domains under-represented in the *L. albipes * lineage are included in this table. Additional columns report the number of domains characterized in each species. Aech: *A. echinatior*, Amel: *A. mellifera*, Cflo: *C. floridanus*, Dmel: *D. melanogaster*, Hsal: *H. saltator*, Lalb: *L. albipes*, Nvit: *N. vitripennis*, Sinv: *S. invicta*. Click here for file

Additional file 13 **Phylogenetic tree of *yellow *and *MRJP *genes.** The MRJP genes are highlighted in light green (top), *yellow* genes highlighted in light blue (bottom). Red branches are *A. mellifera* orthologs, and dark blue branches are *L. albipes*. Click here for file

Additional file 14 **Putative DNMT homologs in *L. albipes. ***Putative DNMT homologs in *L. albipes* were identified using a BLASTP search against human, chicken, *Nasonia*, and honey bee (*A. mellifera*). *L. albipes* gene IDs, the target ID, and the E-values are included in this table. Click here for file

Additional file 15 **Maximum likelihood tree of DNMT orthologs.** A BLASTP query of the putative dnmt homologs of *L. albipes* (Lalb) to human (Hsap), honey bee (Amel), chicken (Ggal), *Nasonia* (Nvit), and *Drosophila* (Dmel) revealed four *L. albipes* genes that are putative DNA methyltransferases. A maximum-likelihood tree depicts the relationships among the three DNMTs and their respective orthologs in each species. Bootstrap values indicate level of support at each node. Click here for file

Additional file 16 **Distribution of GC content in *L. albipes. ****L. albipes* exons are G+C enriched compared to the genomic background, while introns have lower G+C contents compared to the genome. Click here for file

Additional file 17 **CpG and GpC O/E ratios are negatively correlated.** (A) CpG O/E and (B) GpC O/E are strongly negatively correlated with G+C contents. Consequently, CDs exhibit lower GpC O/E compared to the genomic background. Click here for file

Additional file 18 **CpG and GpC O/E ratios by GC content.** Genes and genomic fragments were divided into five groups according to their G+C content. Our results show that across all the groups, CpG O/E values of CDS are still significantly lower than that of the genome background when GC content is minimized, while GpC O/E values of CDS are highly similar to those of genome background. Click here for file

Additional file 19 **Candidate genes for methylation.** A total of 1,801 genes have significantly lower CpG O/E ratios than the genomic background but not significantly different GpC O/E (FDR <0.2). These represent strong candidates for DNA methylation. GeneID names, CpG O/E, GpC O/E, and FDR-corrected *P* values are included in this table. Click here for file

Additional file 20 **Genes showing signatures of accelerated evolution in *L. albipes. ***Genes showing signatures of accelerated evolution in *L. albipes* relative to other tested lineages. Null omega is the expected omega value; *L. albipes* alternative omega is the estimated omega value for the *L. albipes* lineage as compared to the other tested lineages. Click here for file

Additional file 21 **Genes showing signatures of accelerated evolution in Apoidea.** Genes showing signatures of accelerated evolution in Apoidea (bees) relative to other tested lineages. Null omega is the expected omega value; Apoidea alternative omega is the estimated omega value for the Apoidea branches as compared to the other tested lineages. Click here for file

Additional file 22 **GO enrichment of genes undergoing accelerated evolution in *L. albipes. ***Results of Gene Ontology analyses for genes experiencing accelerated evolution in *L. albipes*. BP: biological process, CC: cellular component, MF: molecular function. Click here for file

Additional file 23 **IPR enrichment of genes experiencing accelerated evolution in *L. albipes. ***IPR enrichment analysis results with IPR IDs and titles for genes experiences accelerated evolution in *L. albipes* relative to other tested lineages. Click here for file

Additional file 24 **KEGG pathway enrichment genes undergoing accelerated evolution in *L. albipes. ***KEGG analysis revealed several pathways associated with genes experiencing accelerated evolution in the *L. albipes* lineage. MapID and Map Title are specified according to the KEGG database. Click here for file

Additional file 25 **Individual resequencing.** Ka/Ks calculations using genome sequences for a solitary and social female identified six genes that appear to be experiencing positive selection between social forms (FDR <0.1). These genes, the length of the coding sequence, synonymous (Ks) and non-synonymous (Ka) substitutions, and their ratio (Ka/Ks) are summarized in this table. Click here for file
