# Erratum to: The draft genome of a socially polymorphic halictid bee, *Lasioglossum albipes*

**DOI:** 10.1186/s13059-014-0574-0

**Published:** 2015-02-12

**Authors:** Sarah D Kocher, Cai Li, Wei Yang, Hao Tan, Soojin V Yi, Xingyu Yang, Hopi E Hoekstra, Guojie Zhang, Naomi E Pierce, Douglas W Yu

**Affiliations:** Department of Organismic and Evolutionary Biology, Museum of Comparative Zoology, Harvard University, 26 Oxford St, Cambridge, MA 02138 USA; China National GeneBank, BGI-Shenzhen, Shenzen, 518083 China; Centre for GeoGenetics, Natural History Museum of Denmark, University of Copenhagen, Øster Voldgade 5-7, Copenhagen, 1350 Denmark; School of Biology, Georgia Institute of Technology, Atlanta, GA 30332 USA; Department of Molecular and Cellular Biology, Howard Hughes Medical Institute, Harvard University, 26 Oxford St, Cambridge, MA 02138 USA; Centre for Social Evolution, Department of Biology, University of Copenhagen, Universitetsparken 15, Copenhagen, DK-2100 Denmark; State Key Laboratory of Genetic Resources and Evolution, Kunming Institute of Zoology, Kunming, Yunnan 650223 China; School of Biological Sciences, University of East Anglia, Norwich Research Park, Norwich, Norfolk NR47TJ UK

## Abstract

During the type-setting of the final version of the article [1] some of the additional files were swapped, and several were completely replaced. The correct files are republished in this Erratum.

**Electronic supplementary material:**

The online version of this article (doi:10.1186/s13059-014-0574-0) contains supplementary material, which is available to authorized users.

## Erratum

During the type-setting of the final version of the article [1] some of the additional files were swapped, and several were completely replaced. The editors apologize for the clerical mistake that led to the loss of some of the additional files.

In the final version of the article:

Additional File 8 is wrongly denoted as Additional File 22;

Additional File 9 is wrongly denoted as Additional File 23;

Additional File 10 is wrongly denoted as Additional File 24;

Additional File 11 is wrongly denoted as Additional File 25;

Additional File 12 is wrongly denoted as Additional File 1;

Additional File 14 is wrongly denoted as Additional File 3;

Additional File 20 is wrongly denoted as Additional File 9;

Additional File 21 is wrongly denoted as Additional File 10;

Additional File 22 is wrongly denoted as Additional File 11;

Additional File 23 is wrongly denoted as Additional File 12;

Additional File 25 is wrongly denoted as Additional File 14;

Additional Files 1 is wrongly replaced by Additional File 12;

Additional Files 3 is wrongly replaced by Additional File 14;

Additional Files 5 is wrongly replaced by Additional File 16;

Additional Files 24 is wrongly replaced by Additional File 10.

Additional Files 2, 4, 6, 7, 13, 15, 16, 17, 18 and 19 are correct in the final version of the article.

All Additional Files were correct in the provisional version of the article. Below please find the correct full list of Additional Files associated with this article.

## Additional files

Additional file 1:
**Sample information.** Sample collection data for specimens used in genome and transcriptome sequencing. Sample names, sex, collection dates, region, and GPS coordinates are specified, as well as the libraries each specimen was used to construct. Additional file 2:
**Repeats in the genome.** Repeat annotation was conducting using RepeatMasker. The overlaps between repeats have been excluded before the calculation of the total size. The length and percent of the genome comprised by each repeat is included. Additional file 3:
**Genome assembly comparisons.** Comparison of genome assemblies for sequenced hymenopteran species. *L. albipes* is highly comparable to these other sequenced species. Additional file 4:
**Gene prediction statistics.** Gene prediction relied on three strategies: *de novo* prediction, homology-based approaches using four well-annotated genomes, and RNA sequencing (CCG). Statistics indicate the number of genes annotated with each method, the average transcript and coding sequence (CDS) lengths, the average number of exons per gene, and the average exon and intron lengths. Additional file 5:
**Gene predictions in comparison to other sequenced insect genomes.** Comparisons of coding sequence (CDS), mRNA, exon, and intron length were conducted across five arthropod genomes. Amel: *Apis mellifera,* Cele: *Caenorhabditis elegans,* Dmel: *Drosophila melanogaster,* Hsal: *Harpegnathos saltator*, Lalb: *Lasioglossum albipes.*
Additional file 6:
**Orthology between** ***L. albipes*** **and other species.** The top row includes the number of genes annotated in the current *L. albipes* assembly, and subsequent rows represent the number of orthologs in *L. albipes* in comparison with each named species, all sequenced ants (*H. saltator, C. floridanus, A. echinatior, S. invicta, L. humile, P. barbatus,* and *A. cephalotes*), and all sequenced Hymenoptera (all ants plus *A. mellifera* and *N. vitripennis*). Additional file 7:
**Non-coding RNA genes in the genome.** Annotated ncRNA summary statistics. The average length of miRNA is for the predicted precursor miRNA. The number of copies annotated in the genome, their average length in basepairs, summed total length, and the percentage of the genome comprised by each element are included. Additional file 8:
**GO enrichment in** ***L. albipes*** **specific genes.** The *P* values were adjusted by FDR and the cutoff of adjusted *P* value is 0.05. Additional file 9:
**IPR enrichment in** ***L. albipes*** **specific genes.** The *P* values were adjusted by FDR and the cutoff of adjusted *P* value is 0.05. Additional file 10:
**IPR domains over-represented in the** ***L. albipes*** **lineage.** The domains that have at least 10 copies are included in this table. Additional columns report the number of domains characterized in each species. Aech: *A. echinatior,* Amel: *A. mellifera*, Cflo: *C. floridanus*, Dmel: *D. melanogaster*, Hsal: *H. saltator*, Lalb: *L. albipes*, Nvit: *N. vitripennis*, Sinv: *S. invicta*. Additional file 11:
**Putatively lost genes in** ***L. albipes*** **lineage.** Genes that appear to be lost in the *L. albipes* lineage are included in this table. The functions are derived from Swiss-Prot annotation database. *Amel* gene IDs represent the gene annotation symbol in the *Apis mellifera* genome assembly. Additional file 12:
**IPR domains under-represented in** ***L. albipes*** **lineage.** IPR domains under-represented in the *L. albipes* lineage are included in this table. Additional columns report the number of domains characterized in each species. Aech: *A. echinatior*, Amel: *A. mellifera*, Cflo: *C. floridanus*, Dmel: *D. melanogaster*, Hsal: *H. saltator*, Lalb: *L. albipes*, Nvit: *N. vitripennis*, Sinv: *S. invicta*. Additional file 13:
**Phylogenetic tree of** ***yellow*** **and** ***MRJP*** **genes.** The MRJP genes are highlighted in light green (top), *yellow* genes highlighted in light blue (bottom). Red branches are *A. mellifera* orthologs, and dark blue branches are *L. albipes*. Additional file 14:
**Putative DNMT homologs in** ***L. albipes.*** Putative DNMT homologs in *L. albipes* were identified using a BLASTP search against human, chicken, *Nasonia*, and honey bee (*A. mellifera*). *L. albipes* gene IDs, the target ID, and the E-values are included in this table. Additional file 15:
**Maximum likelihood tree of DNMT orthologs.** A BLASTP query of the putative dnmt homologs of *L. albipes* (Lalb) to human (Hsap), honey bee (Amel), chicken (Ggal), *Nasonia* (Nvit), and *Drosophila* (Dmel) revealed four *L. albipes* genes that are putative DNA methyltransferases. A maximum-likelihood tree depicts the relationships among the three DNMTs and their respective orthologs in each species. Bootstrap values indicate level of support at each node. Additional file 16:
**Distribution of GC content in** ***L. albipes.*** *L. albipes* exons are G+C enriched compared to the genomic background, while introns have lower G+C contents compared to the genome. Additional file 17:
**CpG and GpC O/E ratios are negatively correlated.** (A) CpG O/E and (B) GpC O/E are strongly negatively correlated with G+C contents. Consequently, CDs exhibit lower GpC O/E compared to the genomic background. Additional file 18:
**CpG and GpC O/E ratios by GC content.** Genes and genomic fragments were divided into five groups according to their G+C content. Our results show that across all the groups, CpG O/E values of CDS are still significantly lower than that of the genome background when GC content is minimized, while GpC O/E values of CDS are highly similar to those of genome background. Additional file 19:
**Candidate genes for methylation.** A total of 1,801 genes have significantly lower CpG O/E ratios than the genomic background but not significantly different GpC O/E (FDR <0.2). These represent strong candidates for DNA methylation. GeneID names, CpG O/E, GpC O/E, and FDR-corrected *P* values are included in this table. Additional file 20:
**Genes showing signatures of accelerated evolution in** ***L. albipes.*** Genes showing signatures of accelerated evolution in *L. albipes* relative to other tested lineages. Null omega is the expected omega value; *L. albipes* alternative omega is the estimated omega value for the *L. albipes* lineage as compared to the other tested lineages. Additional file 21:
**Genes showing signatures of accelerated evolution in Apoidea.** Genes showing signatures of accelerated evolution in Apoidea (bees) relative to other tested lineages. Null omega is the expected omega value; Apoidea alternative omega is the estimated omega value for the Apoidea branches as compared to the other tested lineages. Additional file 22:
**GO enrichment of genes undergoing accelerated evolution in** ***L. albipes.*** Results of Gene Ontology analyses for genes experiencing accelerated evolution in *L. albipes*. BP: biological process, CC: cellular component, MF: molecular function. Additional file 23:
**IPR enrichment of genes experiencing accelerated evolution in** ***L. albipes.*** IPR enrichment analysis results with IPR IDs and titles for genes experiences accelerated evolution in *L. albipes* relative to other tested lineages. Additional file 24:
**KEGG pathway enrichment genes undergoing accelerated evolution in** ***L. albipes.*** KEGG analysis revealed several pathways associated with genes experiencing accelerated evolution in the *L. albipes* lineage. MapID and Map Title are specified according to the KEGG database. Additional file 25:
**Individual resequencing.** Ka/Ks calculations using genome sequences for a solitary and social female identified six genes that appear to be experiencing positive selection between social forms (FDR <0.1). These genes, the length of the coding sequence, synonymous (Ks) and non-synonymous (Ka) substitutions, and their ratio (Ka/Ks) are summarized in this table.
