# Supplementary figures and images for: The draft genome of a socially polymorphic halictid bee, Lasioglossum albipes
Source: Genome Biol. 2013 Dec 20;14(12):R142. doi: 10.1186/gb-2013-14-12-r142 (PMC4062844; doi:10.1186/gb-2013-14-12-r142)

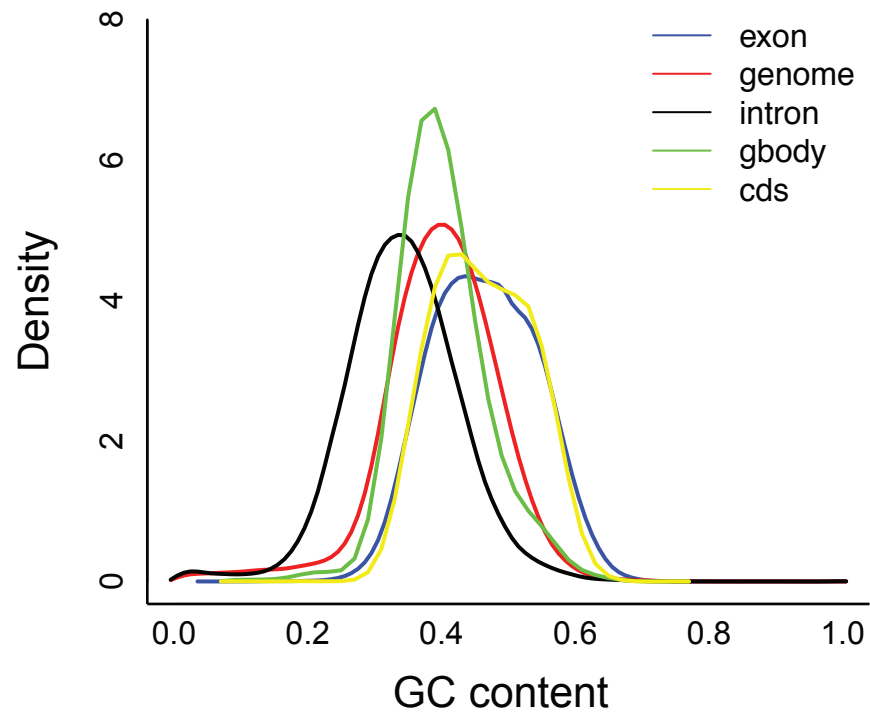

Figure S15

Supplement: Additional file 5 — Gene predictions in comparison to other sequenced insect genomes. Comparisons of coding sequence (CDS), mRNA, exon, and intron length were conducted across five arthropod genomes. Amel: Apis mellifera, Cele: Caenorhabditis elegans, Dmel: Drosophila melanogaster, Hsal: Harpegnathos saltator, Lalb: Lasioglossum albipes. [file gb-2013-14-12-r142-S5.pdf]

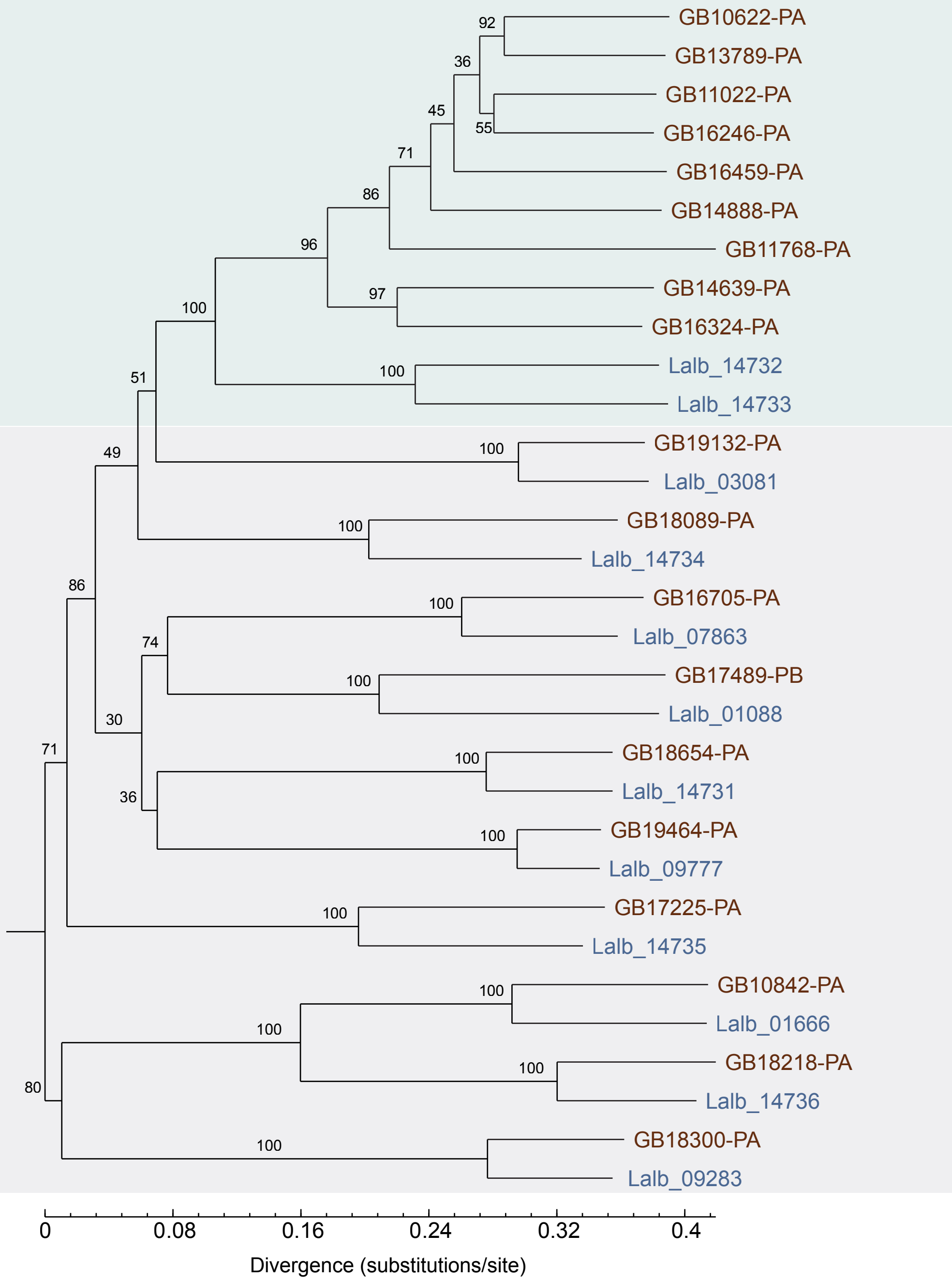

Figure S12

Supplement: Additional file 13 — Phylogenetic tree of yellow and MRJP genes. The MRJP genes are highlighted in light green (top), yellow genes highlighted in light blue (bottom). Red branches are A. mellifera orthologs, and dark blue branches are L. albipes. [file gb-2013-14-12-r142-S13.pdf]

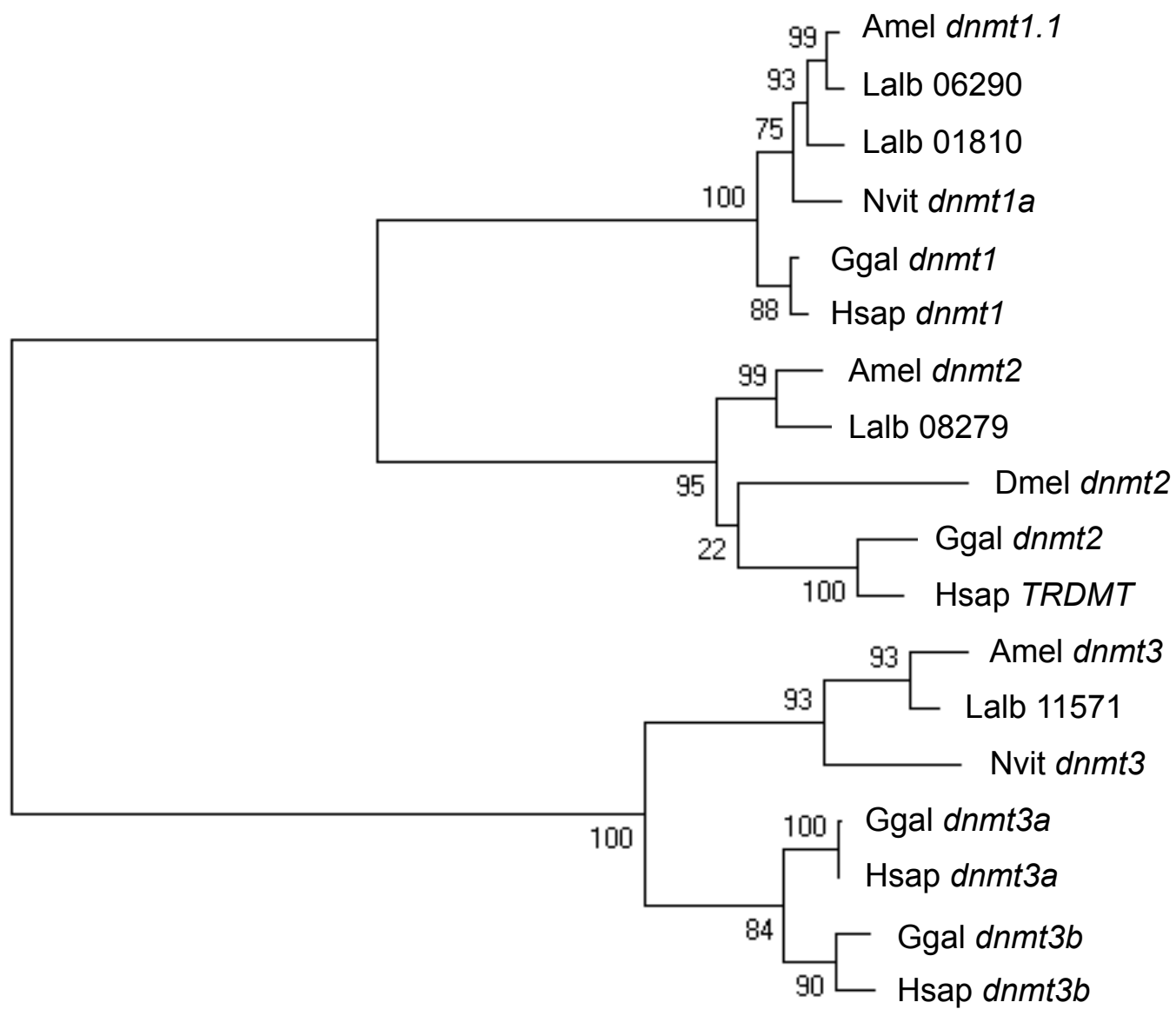

Figure S14

Supplement: Additional file 15 — Maximum likelihood tree of DNMT orthologs. A BLASTP query of the putative dnmt homologs of L. albipes (Lalb) to human (Hsap), honey bee (Amel), chicken (Ggal), Nasonia (Nvit), and Drosophila (Dmel) revealed four L. albipes genes that are putative DNA methyltransferases. A maximum-likelihood tree depicts the relationships among the three DNMTs and their respective orthologs in each species. Bootstrap values indicate level of support at each node. [file gb-2013-14-12-r142-S15.pdf]

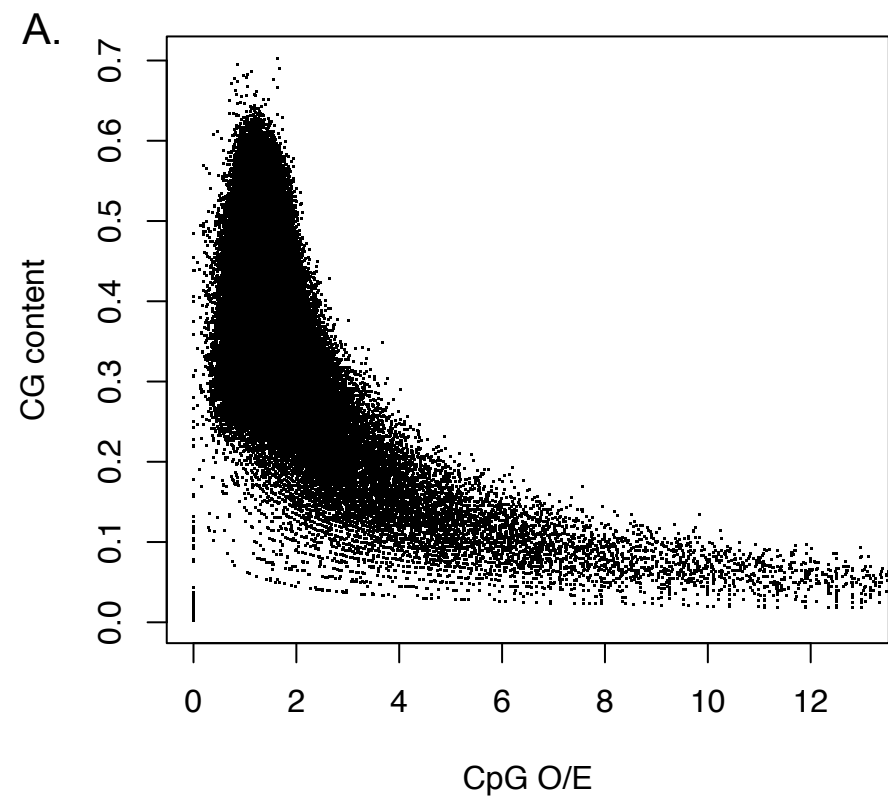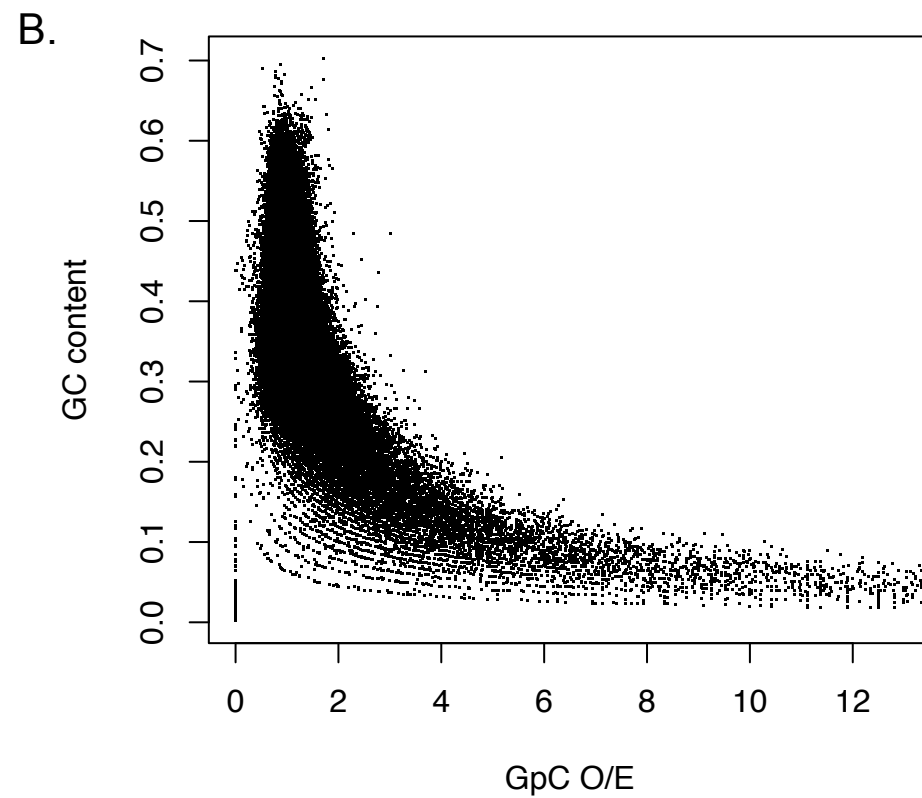

Figure S16

Supplement: Additional file 17 — CpG and GpC O/E ratios are negatively correlated. (A) CpG O/E and (B) GpC O/E are strongly negatively correlated with G+C contents. Consequently, CDs exhibit lower GpC O/E compared to the genomic background. [file gb-2013-14-12-r142-S17.pdf]

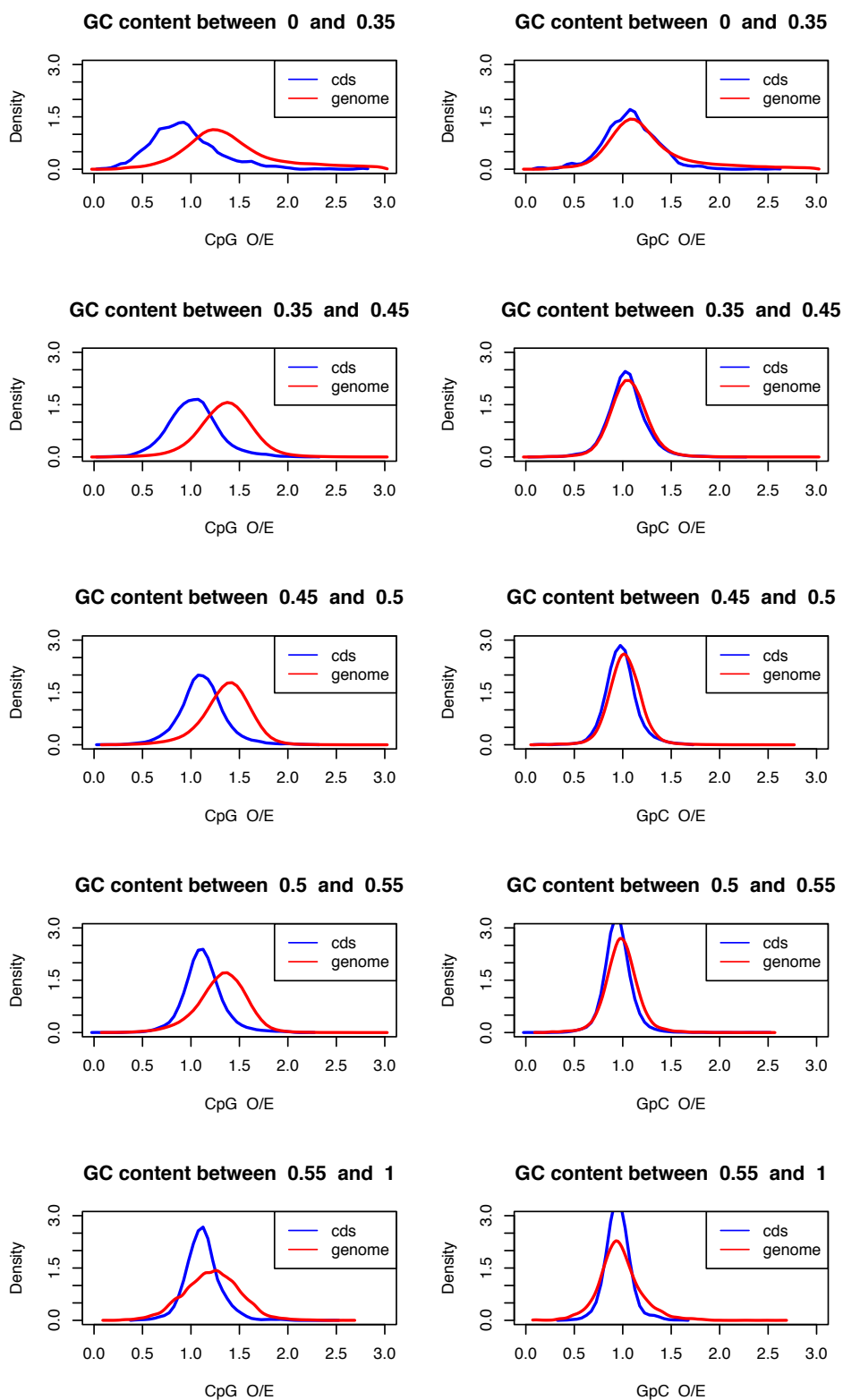

Figure S17

Supplement: Additional file 18 — CpG and GpC O/E ratios by GC content. Genes and genomic fragments were divided into five groups according to their G+C content. Our results show that across all the groups, CpG O/E values of CDS are still significantly lower than that of the genome background when GC content is minimized, while GpC O/E values of CDS are highly similar to those of genome background. [file gb-2013-14-12-r142-S18.pdf]
